# Analysis of Serum microRNA Expression Profiles and Comparison with Small Intestinal microRNA Expression Profiles in Weaned Piglets

**DOI:** 10.1371/journal.pone.0162776

**Published:** 2016-09-15

**Authors:** Xin Tao, Ziwei Xu, Xiaoming Men

**Affiliations:** Institute of Animal Husbandry and Veterinary Science, Zhejiang Academy of Agricultural Sciences, Hangzhou, Zhejiang, China; Kunming University of Science and Technology, CHINA

## Abstract

Weaning stress induces tissue injuries and impairs health and growth in piglets, especially during the first week post-weaning. MicroRNAs (miRNAs) play vital roles in regulating stresses and diseases. Our previous study found multiple differentially expressed miRNAs in small intestine of piglets at four days post-weaning. To better understand the roles of miRNAs during weaning stress, we analyzed the serum miRNA expressional profile in weaned piglets (at four days post-weaning) and in suckling piglets (control) of the same age using miRNA microarray technology. We detected a total of 300 expressed miRNAs, 179 miRNAs of which were differentially expressed between the two groups. The miRNA microarray results were validated by RT-qPCR. The biological functions of these differentially expressed miRNAs were predicted by GO terms and KEGG pathway annotations. We identified 10 highly expressed miRNAs in weaned piglets including miR-31, miR-205, and miR-21 (upregulated) and miR-144, miR-30c-5p, miR-363, miR-194a, miR-186, miR-150, and miR-194b-5p (downregulated). Additionally, miR-194b-5p expression was significantly downregulated in serum and small intestine of weaned piglets. Our results suggest that weaning stress affects serum miRNA profiles in piglets. And serum miR-194b-5p levels can reflect its expressional changes in small intestine of piglets by weaning stress.

## Introduction

Weaning that is one of the most stressful events in a pig’s life impacts animal health and growth performance, especially during the first week post-weaning [1–3]. Weaning stress affects multiple systems in weaned piglets including gastrointestinal, immune, and endocrine systems [4–6]. It is impossible to completely eliminate the adverse physiological effects of weaning stress. However, a thorough understanding of the molecular mechanisms involved during weaning would assist in alleviating stress-induced injuries.

MicroRNAs (miRNAs) play important roles in regulating stress signaling pathways and diseases [7]. Plasma miRNAs are detected for the first time in patients with tumors [8]. And it was demonstrated that there are no significant differences between plasma and serum miRNA levels, and miRNA levels are not affected by high temperatures, pH, or repeated freezing/thawing cycles [9]. Compared with other biological fluids, serum miRNAs are promising and stable biomarkers [10]. In recent years, serum/plasma miRNAs have been used in the diagnoses of tissue injuries [11], arthritis [12], cardiovascular diseases [13], chronic hepatitis [14], diabetes [15], and tumors [16–18].

Our previous study revealed that weaning stress contributes to differentially expressed miRNAs that regulate small intestinal metabolism, stress response, and immune function in piglets [19]. In this study, we assessed whether weaning stress affects expression of serum miRNAs and the association between miRNA expression in serum and miRNA expression in small intestine of piglets.

## Materials and Methods

### Sample collection

Animal studies were conducted in accordance with the principles and guidelines of the Zhejiang Farm Animal Welfare Council of China and were approved by the ethics committee of Zhejiang Academy of Agricultural Sciences.

Animal treatments have been described elsewhere [19]. Prior to slaughter, blood samples were collected into 10 ml EDTA-containing tubes from the jugular vein of four suckling piglets (29 days of age, control) and four weaned piglets (four days post-weaning, 29 days of age). Blood samples were centrifuged at 3,500 g for 10 min at 4°C. The resulting serum samples were stored at -80°C.

### Total RNA isolation and quality control

Total RNA was extracted from serum samples using miRNeasy mini kit (Qiagen, Toronto, ON, Canada). Total RNA concentration was determined in a NanoDrop1000 (Thermo Fisher Scientific, Wilmington, DE, USA). RNA quality was assessed by electrophoresis on a formaldehyde-containing, ethidium bromide-stained 1% (w/v) agarose gel, and RNA integrity was evaluated on an Agilent 2100 Bioanalyzer (Agilent Technologies, Santa Clara, CA, USA).

### Microarrays

Microarrays were performed by LC Sciences (Houston, TX, USA). The microarray design was based on porcine miRNA mature sequences of legumes downloaded in miRBase Release 20.0 (http://www.mirbase.org/) and 100 characterized miRNAs [19]. Each probe was repeated eight times on the chip to ensure reproducibility of the microarray. For the assay, 5 μg of total RNA was used from each sample. Total RNA was size fractionated through a YM 100 microcon filter (Millipore, Bedford, MA, USA); small RNAs (< 300 nucleotides) were 3’-extended with a poly (A) tail using poly (A) polymerase. Subsequently, an oligonucleotide tag was ligated to the poly (A) tail for fluorescent dye staining. Hybridization was performed overnight on a μParaflo microfluidic chip using a micro-circulation pump (Atactic Technologies, Houston, TX, USA) [20]. On the microfluidic chip, each probe consisted of a chemically modified nucleotide-coding segment complementary to its target miRNA and a spacer segment containing polyethylene glycol to enlarge the encoding segment away from the substrate. The detection probes were in situ synthesized with photogenerated reagent. The hybridization melting temperatures were balanced by chemical modifications of the detection probes. The hybridization process required 100 μl of 6×SSPE buffer (0.90 M NaCl, 60 mM Na_2_HPO_4_, and 6 mM EDTA, pH 6.8) containing 25% formamide at 34°C. Following hybridization detection, fluorescence labeling was performed with tag-conjugating Cy3 dye. Hybridization images were collected with a laser scanner (GenePix 4000B, Molecular Devices, Sunnyvale, CA) and digitized with Array-Pro image analysis software (Media Cybernetics, Silver Spring, MD, USA).

The raw data and processed files for the miRNA chip have been deposited in NCBI Gene Expression Omnibus database and are accessible through GEO Series accession number GSE 69920.

### RT-qPCR

Stem-loop RT-qPCR of RNA samples was performed [21, 22]. ReverTraAce reverse transcriptase (Toyobo, Osaka, Japan) and miRNA-specific stem-loop RT primers were used to synthesize cDNA. Reaction mixtures were incubated at 65°C for 5 min, 37°C for 15 min, and 98°C for 5 min. RT-qPCR was performed using SYBR Green Real-time PCR Master Mix (Toyobo, Osaka, Japan) in an ABI StepOne Plus real-time PCR system (Applied Biosystems, Foster City, CA, USA). Swine U6 snRNA was used as an internal control. All reactions were carried out in triplicate. Relative quantification was calculated using 2^-ΔΔCt^.

### Gene target prediction and functional annotation

Target sites were predicted with miRanda algorithm (http://www.microrna.sanger.ac.uk), TargetScans (http://genes.mit.edu/tscan/targetscanS2005.html), and PicTar (http://pictar.org/). GO terms were enriched with GOEAST software. GO functional enrichment of differentially expressed miRNAs was categorized using level 2 (q value was the adjusted p value of the test statistic) [23]. KEGG pathway annotations were performed using DAVID gene annotation tool; significance was set at q < 0.05 (q value was the adjusted p value of the test statistic).

### Statistical analyses

Microarray data were analyzed after subtracting the background. Microarray signals were normalized using LOWESS filter (Locally-Weighted Regression) [24]. To be considered a differentially expressed miRNA, the hybridization signal had to be ≥ 500 at p < 0.01.

RT-qPCR data were analyzed by Student’s t-test using SPSS version 17.0 statistical software. The results were expressed as mean ± SEM. Statistical significance was set at p < 0.05.

## Results

### Microarray analyses

**Fig 1:**
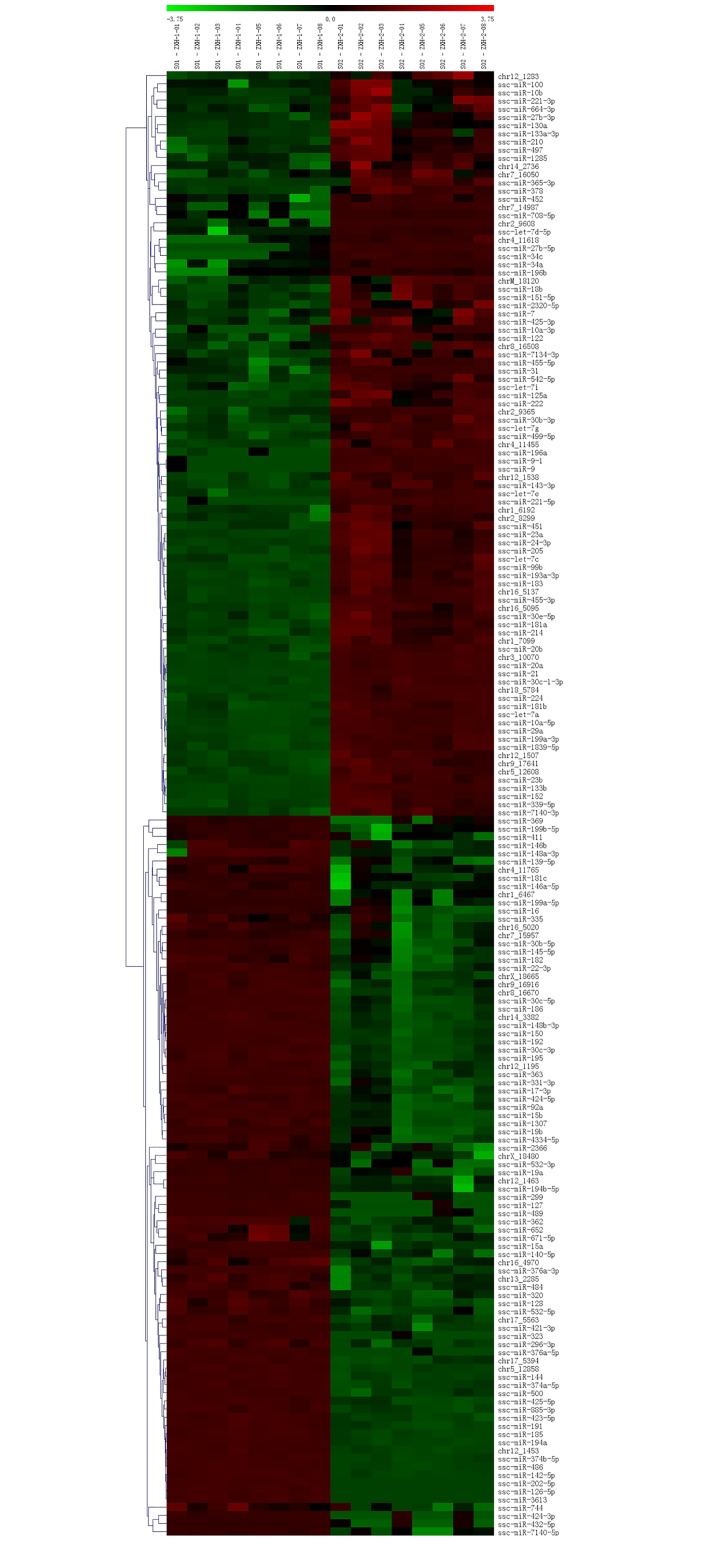
Heatmap of differentially expressed miRNAs in suckling and weaned piglets. Green indicates downregulated expression, and red indicates upregulated expression compared to a reference expression level.

The miRNA chips contained probes for 422 miRNAs (S1 Table) including all 322 porcine miRNAs from the miRBase Release 20.0 and 100 miRNAs previously identified by Solax high-throughput sequencing technology. We detected a total of 300 miRNAs in serum of both weaned and suckling (control) piglets (S2 Table). The microarray results showed that a total of 179 miRNAs were differentially expressed (p < 0.01, Fig 1 and S2 Table). Out of 179 miRNAs, 91 were upregulated and 88 were downregulated in the weaned group. To determine which core miRNAs play key roles during weaning, we set the screening conditions as follows, signal ≥ 2,000 and |log _2_ Fold change|≥ 4 at p < 0.01. We detected 10 core miRNAs in the weaned group: 3 miRNAs (miR-21, miR-31, and miR-205) were upregulated, and 7 miRNAs (miR-30c-5p, miR-144, miR-150, miR-186, miR-194a, miR-194b-5p and miR-363) were downregulated.

### Validation of miRNA expression via stem-loop RT-qPCR

**Fig 2:**
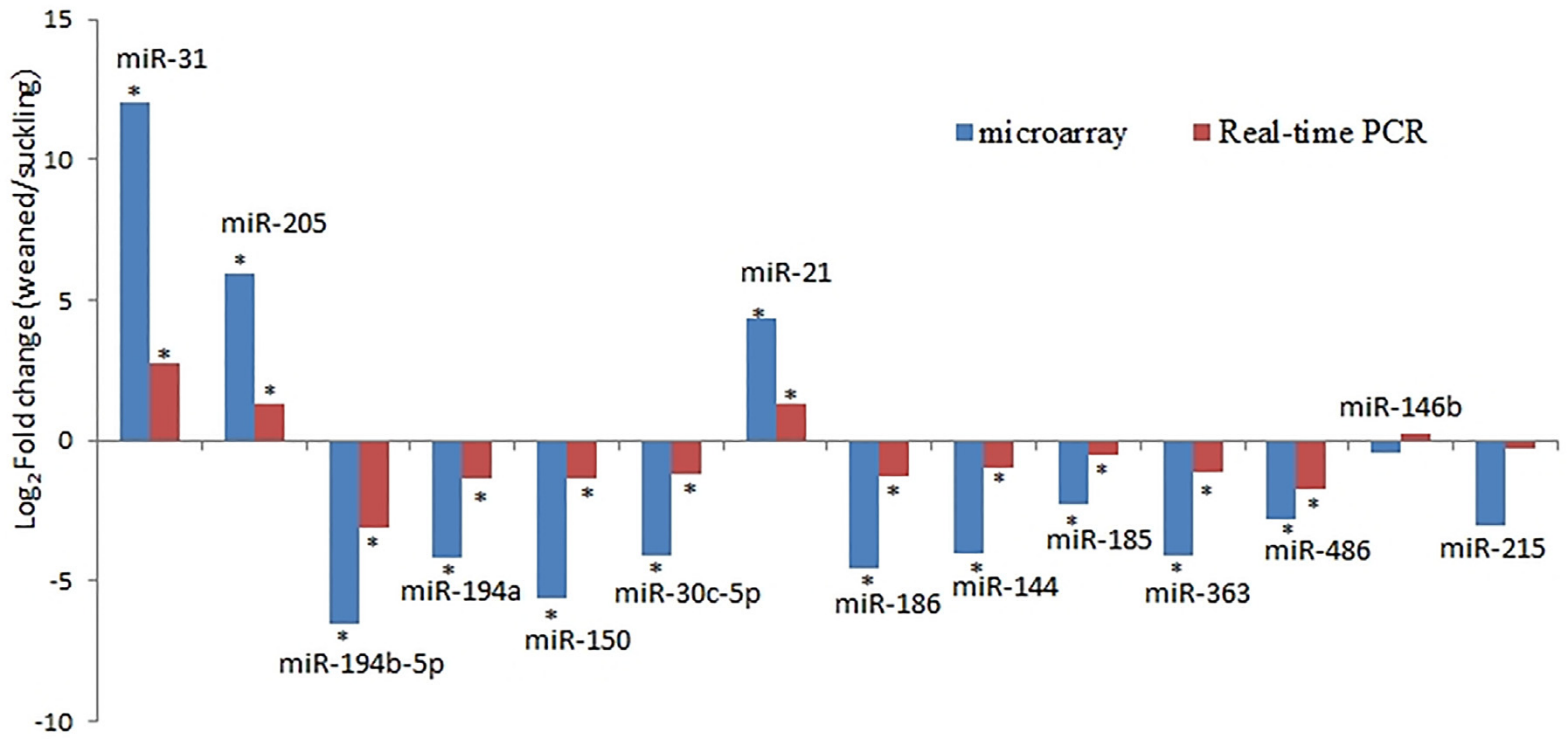
Validation of microarray results by RT qPCR. Four biological replicates were used, and U6 snRNA was used as an internal control. Means with the star on the column differ significantly (P<0.01 for microarray; P<0.05 for RT qPCR).

To validate the microarray results, we quantified the expression of 14 miRNAs by RT-qPCR. In addition to 10 core miRNAs, miR-185 and miR-486 were selected due to the abundant expression levels in suckling piglets and |log _2_ Fold change|≥ 2 at p < 0.01; we are also interested in miR-215 and miR-146b because the former showed the abundant expression level and the latter had the largest differences in expression levels in small intestine of piglets [19]. The miRNA primers are presented in S3 Table. The expression levels of these miRNAs obtained from microarray and RT-qPCR were compared (Fig 2). The expression levels of 13 out of 14 miRNAs, including 10 core miRNAs, were similar between microarray and RT-qPCR. On the other hand, RT-qPCR results revealed that the expression level of miR-146b was upregulated compared to the microarray results; however, the difference was not significant (p > 0.05). This result may have been attributed to differences in the sensitivity of the two methods.

### MiRNA target gene prediction and functional annotation

The potential target sites of differentially expressed miRNAs between suckling and weaned piglets were predicted by miRanda algorithm, TargetScans, and PicTar software. A total of 5,398 miRNA-mRNA interaction sites corresponding to 565 target genes were predicted (S4 Table). All miRNAs regulated more than one target. On the other hand, most of the target sites were regulated by more than one miRNA, and < 12% genes had only one special target site. By contrast, three genes (*GOLT1B*, *EEA1*, and *HCFC2*) were assigned the highest number of miRNAs.

We evaluated the biological functions of differentially expressed miRNAs by performing GO terms and KEGG pathway annotations. The lists of the pathways ranked by enrichment are presented in S5 and S6 Tables. To these target genes of differentially expressed miRNAs, GO function enrichment revealed that they were involved in 42 enriched biological functions including stress response, antigen processing and presentation, and immune response, etc. KEGG pathway analysis included 25 pathways regulating cancer, the chemokine signaling pathway, the PPAR signaling pathway, and the intestinal immune network for IgA production, etc.

### Analysis of differentially expressed miRNAs between serum and small intestine

To assess whether changes in serum miRNAs can reflect changes in intestinal miRNAs, we compared 10 core miRNAs differentially expressed in serum with those in the small intestine (S7 Table) previously obtained. The results showed that changes in miR-194b-5p (downregulated) and miR-205 (upregulated) were consistent between serum and small intestinal samples of weaned piglets. Moreover, miR-194b-5p had the highest expression level amongst all differentially expressed miRNAs and was one of the few downregulated miRNAs in small intestine of weaned piglets. On the contrary, miR-150 and miR-363 were upregulated; the remaining 6 miRNAs (miR-21, miR-30c-5p, miR-31, miR-144, miR-186 and miR-194a) had no significant differences in small intestine.

**Fig 3:**
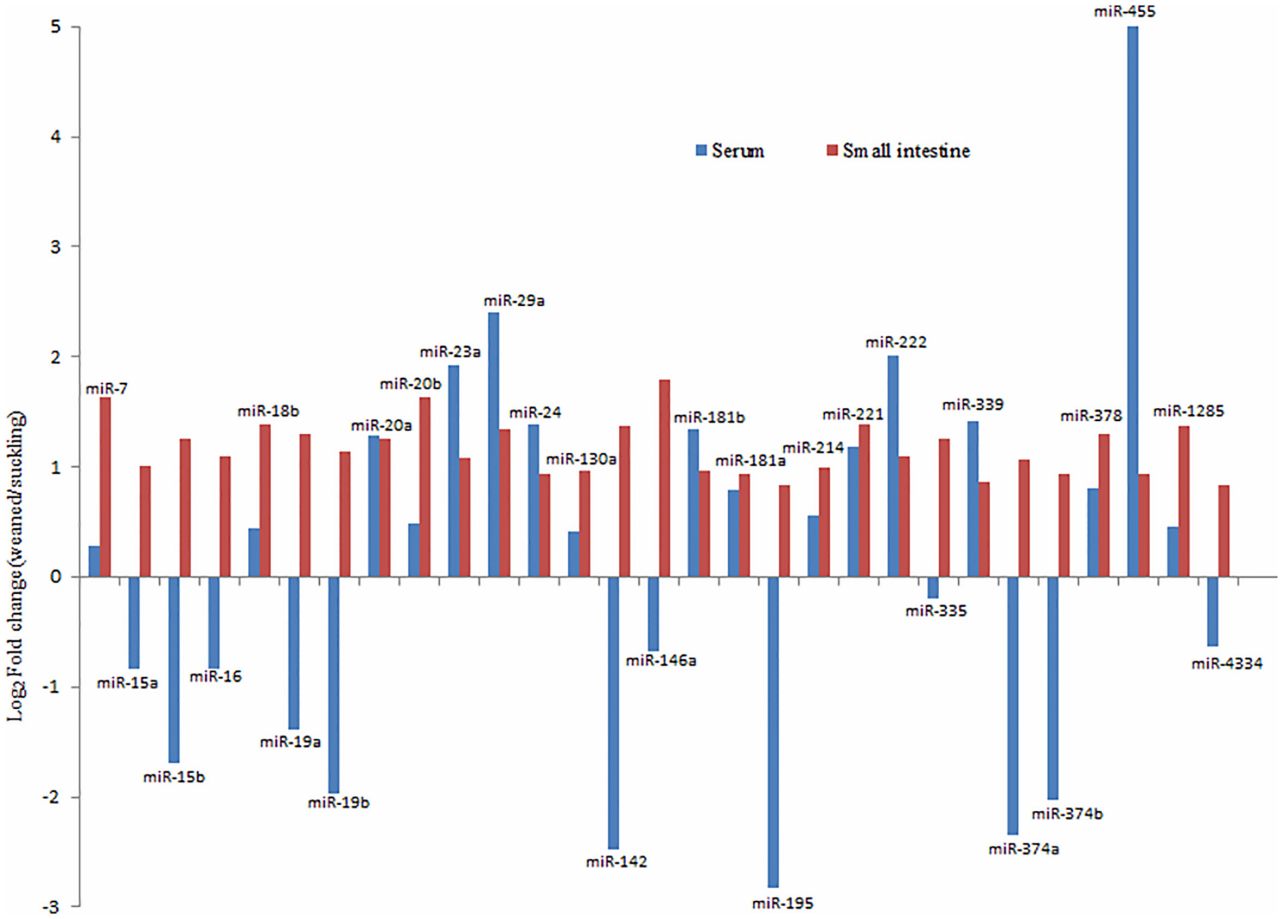
Comparison of all differentially expressed miRNAs (excluding 10 core miRNAs) in serum with those in small intestine.

In addition to 10 core miRNAs, we further compared all differentially expressed miRNAs (signal ≥ 500 at p < 0.01) in serum with those in small intestine. The results showed a total of 29 known miRNAs presented differential expression both in serum and small intestine (Fig 3). Out of 29 miRNAs, 17 were consistent and all upregulated in the weaned group; the remaining 12 were downregulated in serum but upregulated in small intestine. Furthermore, according to the expressional differences (|log _2_ Fold change|≥ 1) both in serum and the small intestine, only 5 (miR-20a, miR-23a, miR-29a, miR-221 and miR-222) of 17 miRNAs in serum may reflect the expressional changes of themselves in small intestine. However, the results of their miRNA chips (serum) and miRNA high-throughput sequencing (the small intestine) still need to be verified by RT-qPCR.

## Discussion

Studies have shown that changes in serum miRNA levels are linked to pathological stress and diseases. In certain diseases, miRNAs can be quickly released from injured tissues into body fluids [25]. Even though emerging studies have revealed a correlation between serum/plasma miRNAs and different pathological conditions in humans [26–29], there is little information on porcine serum/plasma miRNAs. Our study results showed that there are lots of miRNAs in porcine serum and more than 100 dysregulated miRNAs in weaned piglets. These differentially expressed miRNAs are probably attributed to weaning stress. Among 10 core miRNAs, miR-21 had the highest expression in weaned piglets. In addition to its role in tumor promotion as an oncomiR, miR-21 participates in the pathology of stress [30]. Recent data have shown that miR-21 regulates the immune system, antiviral response, and traumatic brain injury [31–33]. Human plasma/serum miR-21 is associated with inflammation, cardiovascular disease, obesity, and kidney fibrosis [34–37]. Therefore, in this study, upregulated serum miR-21 may be indicative of tissue injury and depressed immune function in weaned piglets. Our study findings revealed that miR-205, which is highly expressed in medullary thymic epithelial cells [38], was upregulated in serum of weaned piglets. Notably, the weight of thyroid glands decreases by 55.74% at seven days post-weaning [39]. Therefore, there is likely to be an association between miR-205 and the changes of thyroid gland induced by weaning stress. Downregulated miR-150 expression is associated with inflammation and bacterial infections. MiR-150 represents a key regulator of immune cell differentiation and activation; LPS injections reduce expression levels of miR-150 in leukocytes of healthy human [40–42]. Furthermore, low serum miR-150 levels correlated with hepatic and/or renal dysfunction [43, 44]. More intriguingly, a recent study showed that plasma miR-144 levels were lower in patients with depression than in healthy controls. Following treatment, miR-144 levels increased [45]. Piglets suffer from seriously psychological stress when there is a sudden separation from the sow and are exposed to a different environment. It is possible that serum miR-144 levels might reflect the psychological state of weaned piglets. Studies on the other miRNAs, i.e., miR-31, miR-186, miR-30c-5p, and miR-363, have focused on their association to tumor and cancer.

It has been demonstrated miR-185 plays an important role in regulating fatty acid metabolism, cholesterol homeostasis and insulin signaling [46]. MiR-185 expression levels were increased in mice fed a high-fat diet, and this increase was correlated with an increase in total cholesterol level [47]. And plasma miR-185 was significantly decreased in diabetic patients and mice [48]. Notably, serum miR-486 was also identified to be in association with some lipid metabolism biomarkers in coronary artery disease patients [49]. In healthy young men, serum miR-486 was significantly decreased due to acute or chronic exercise. And the reduction may be associated with metabolic changes during exercise and adaptation induced by training [50]. In our study, serum miR-185 and miR-486 presented abundant expression in suckling piglets. However, their levels were significantly decreased in weaned piglets. These findings suggested miR-185 and miR-486 may regulate the dysfunction of metabolism induced by weaning stress.

Although our previous study found that miR-215 had the abundant expression level and miR-146b presented the largest differences in expression levels in the small intestine of piglets [19], and they were demonstrated to regulate the growth and development of gastrointestinal tract [51, 52], in this study both serum miR-215 and miR-146b had lower expression levels, and no significant differences have been found between weaned and suckling piglets.

It has been reported that miR-194 is highly expressed in the small intestine of piglets [53]. In our previous study, miR-194b-5p was expressed in the small intestine and was downregulated in weaned piglets [19]. In the present study, we found that serum miR-194b-5p levels of weaned piglets was significantly reduced compared to those of suckling piglets, probably due to decreased miR-194b-5p levels in the intestine of weaned piglets. Moreover, miR-194, which is highly induced during intestinal epithelium differentiation, plays an important role in intestinal epithelium maturation [54]. Serum miR-194b-5p may be a potential biomarker of small intestine injury in weaned piglets.

The GO analysis of the differentially expressed miRNAs illustrated that a high enrichment of GOs was involved in membrane-bounded organelles, single-organism cellular processes, biological process regulation, ion binding, organic substance metabolic processes, primary metabolic processes, and cellular metabolic processes. Furthermore, GOs of stress response, antigen processing and presentation, and immune response were significantly enriched. Analyses of the KEGG pathway identified 25 pathways, in addition to cancer-related pathways, which included the chemokine signaling pathway, cell adhesion molecules, the cell cycle and hematopoietic cell lineage, the PPAR signaling pathway, chronic myeloid leukemia, and the intestinal immune network for IgA production. Our findings revealed that these differentially expressed miRNAs were probably involved in regulating porcine immune functions and stress responses during weaning.

## Conclusions

In summary, we observed 179 differentially expressed miRNAs in serum of weaned piglets. The results of GO terms and KEGG pathway annotations partially identified the potential functions of these differentially expressed miRNAs. We also identified 10 highly expressed miRNAs in weaned piglets, which were probably closely related to weaning stress-induced injury, including miR-31, miR-205, and miR-21 (upregulated) and miR-144, miR-30c-5p, miR-363, miR-194a, miR-186, miR-150, and miR-194b-5p (downregulated). Furthermore, miR-194b-5p might be a potential and noninvasive biomarker of small intestinal injury in weaned piglets.

## Supporting Information

S1 Table Sequences of miRNA probes used for microarray. (XLSX)

S2 Table Serum miRNAs expression profile in suckling andweaned piglets. (XLS)

S3 Table Primer sequences used in RT-qPCR. *The universal reverse primer. ^#^ Also as the reverse primer of U6 snRNA.
(DOCX)

S4 Table Predicted targets of differentially expressed miRNAs. (XLS)

S5 Table Gene Ontology (GO) functional enrichment for potential miRNA targets. (DOCX)

S6 Table Kyoto Encyclopedia of Genes and Genomes (KEGG) pathway annotations for potential miRNA targets. (DOCX)

S7 Table Differentially expressed miRNAs in in the small intestine between suckling and weaned piglets. (XLS)
