# Supplementary material for: Analysis of Serum microRNA Expression Profiles and Comparison with Small Intestinal microRNA Expression Profiles in Weaned Piglets
Source: PLoS One. 2016 Sep 15;11(9):e0162776. doi: 10.1371/journal.pone.0162776 (PMC5025173; doi:10.1371/journal.pone.0162776)
Supplement: S3 Table — *The universal reverse primer. # Also as the reverse primer of U6 snRNA. (DOCX) [file pone.0162776.s003.docx]

**S3 Table. Primer sequences used in RT-qPCR.**

| miRNA ID | Primer | Sequences(5'-3’) |
| --- | --- | --- |
| ssc-miR-21 | RT | GTCGTATCCAGTGCAGGGTCCGAGGTGCACTGGATACGACTCAACAT |
|  | FW | TGCGGTAGCTTATCAGACTGATGTT |
| ssc-miR-31 | RT | GTCGTATCCAGTGCAGGGTCCGAGGTGCACTGGATACGACCAGCTAT |
|  | FW | TGCGGAGGCAAGATGCTGGCATAGC |
| ssc-miR-205 | RT | GTCGTATCCAGTGCAGGGTCCGAGGTGCACTGGATACGACCAGACTC |
|  | FW | TGCGGTCCTTCATTCCACCGGAGTC |
| ssc-miR-194b | RT | GTCGTATCCAGTGCAGGGTCCGAGGTGCACTGGATACGACTCCACAT |
|  | FW | TGCGGTGTAACAGCGACTCCATGTG |
| ssc-miR-150 | RT | GTCGTATCCAGTGCAGGGTCCGAGGTGCACTGGATACGACCACTGGT |
|  | FW | TGCGGTCTCCCAACCCTTGTACCAG |
| ssc-miR-146b | RT | GTCGTATCCAGTGCAGGGTCCGAGGTGCACTGGATACGACGCCTATG |
|  | FW | TGCGGTGAGAACTGAATTCCATAG |
| ssc-miR-144 | RT | GTCGTATCCAGTGCAGGGTCCGAGGTGCACTGGATACGACGTACATC |
|  | FW | TGCGGTACAGTATAGATGATGATGT |
| ssc-miR-30c-5p | RT | GTCGTATCCAGTGCAGGGTCCGAGGTGCACTGGATACGACGCTGAGA |
|  | FW | TGCGGTGTAAACATCCTACACTCTCA |
| ssc-miR-363 | RT | GTCGTATCCAGTGCAGGGTCCGAGGTGCACTGGATACGACTTACAGA |
|  | FW | TGCGGAATTGCACGGTATCCATCTGT |
| ssc-miR-186 | RT | GTCGTATCCAGTGCAGGGTCCGAGGTGCACTGGATACGACAAGCCCT |
|  | FW | TGCGGCAAAGAATTCTCCTTTTGGGC |
| ssc-miR-194a | RT | GTCGTATCCAGTGCAGGGTCCGAGGTGCACTGGATACGACCCACATG |
|  | FW | TGCGGTGTAACAGCAACTCCATGT |
| ssc-miR-215 | RT | GTCGTATCCAGTGCAGGGTCCGAGGTGCACTGGATACGACGTCTGTC |
|  | FW | TGCGGATGACCTATGAATTGACAG |
| ssc-miR-486 | RT | GTCGTATCCAGTGCAGGGTCCGAGGTGCACTGGATACGACCTCGGGG |
|  | FW | TGCGGTCCTGTACTGAGCTGCCCCG |
| ssc-miR-185 | RT | GTCGTATCCAGTGCAGGGTCCGAGGTGCACTGGATACGACTCAGGAA |
|  | FW | TGCGGTGGAGAGAAAGGCAGTTCCT |
|  | URP^*^ | CAGTGCAGGGTCCGAGGT |
| U6 snRNA | RT^#^ | GCTTCGGCAGCACATATACTAAAAT |
|  | FW | CGCTTCACGAATTTGCGTGTCAT |

RT: Reverse transcription primer; FW: Forward primer.

^*^ The universal reverse primer.

^#^ Also as the reverse primer of U6 snRNA.
