# Supplementary material for: Analysis of Serum microRNA Expression Profiles and Comparison with Small Intestinal microRNA Expression Profiles in Weaned Piglets
Source: PLoS One. 2016 Sep 15;11(9):e0162776. doi: 10.1371/journal.pone.0162776 (PMC5025173; doi:10.1371/journal.pone.0162776)
Supplement: S5 Table — (DOCX) [file pone.0162776.s005.docx]

**S5 Table. Gene Ontology (GO) functional enrichment for potential miRNA targets.**

| GO ID | Ontology | Term | Level | Counts | % | q Values |
| --- | --- | --- | --- | --- | --- | --- |
| GO:0044464 | cellular_component | cell part | 2 | 3076 | 60.31372549 | 0.002753579 |
| GO:0043227 | cellular_component | membrane-bounded organelle | 2 | 2027 | 39.74509804 | 8.43093E-36 |
| GO:0044763 | biological_process | single-organism cellular process | 2 | 1890 | 37.05882353 | 6.83603E-22 |
| GO:0050789 | biological_process | regulation of biological process | 2 | 1684 | 33.01960784 | 2.90609E-14 |
| GO:0043167 | molecular_function | ion binding | 2 | 1590 | 31.17647059 | 0.000325808 |
| GO:0071704 | biological_process | organic substance metabolic process | 2 | 1552 | 30.43137255 | 2.80515E-17 |
| GO:0044238 | biological_process | primary metabolic process | 2 | 1488 | 29.17647059 | 1.83694E-16 |
| GO:0044237 | biological_process | cellular metabolic process | 2 | 1343 | 26.33333333 | 1.0136E-16 |
| GO:0005515 | molecular_function | protein binding | 2 | 1141 | 22.37254902 | 3.07593E-20 |
| GO:0044422 | cellular_component | organelle part | 2 | 1057 | 20.7254902 | 2.33709E-10 |
| GO:0044707 | biological_process | single-multicellular organism process | 2 | 856 | 16.78431373 | 1.26681E-06 |
| GO:0036094 | molecular_function | small molecule binding | 2 | 820 | 16.07843137 | 2.94522E-06 |
| GO:0043234 | cellular_component | protein complex | 2 | 783 | 15.35294118 | 7.70335E-09 |
| GO:0044700 | biological_process | single organism signaling | 2 | 714 | 14 | 1.64499E-06 |
| GO:0048856 | biological_process | anatomical structure development | 2 | 705 | 13.82352941 | 3.63192E-07 |
| GO:0006807 | biological_process | nitrogen compound metabolic process | 2 | 686 | 13.45098039 | 1.03649E-08 |
| GO:0016043 | biological_process | cellular component organization | 2 | 662 | 12.98039216 | 2.94522E-06 |
| GO:0044767 | biological_process | single-organism developmental process | 2 | 648 | 12.70588235 | 1.15753E-06 |
| GO:0016740 | molecular_function | transferase activity | 2 | 631 | 12.37254902 | 1.96851E-05 |
| GO:0051234 | biological_process | establishment of localization | 2 | 559 | 10.96078431 | 1.72576E-08 |
| GO:0044710 | biological_process | single-organism metabolic process | 2 | 553 | 10.84313725 | 1.63084E-17 |
| GO:0009058 | biological_process | biosynthetic process | 2 | 526 | 10.31372549 | 0.02769433 |
| GO:0065008 | biological_process | regulation of biological quality | 2 | 488 | 9.568627451 | 6.05466E-13 |
| GO:0006950 | biological_process | response to stress | 2 | 469 | 9.196078431 | 1.39283E-11 |
| GO:0042221 | biological_process | response to chemical stimulus | 2 | 424 | 8.31372549 | 1.15822E-12 |
| GO:0033036 | biological_process | macromolecule localization | 2 | 302 | 5.921568627 | 7.89268E-05 |
| GO:0065009 | biological_process | regulation of molecular function | 2 | 302 | 5.921568627 | 0.003574201 |
| GO:0009056 | biological_process | catabolic process | 2 | 289 | 5.666666667 | 0.000177795 |
| GO:0016491 | molecular_function | oxidoreductase activity | 2 | 283 | 5.549019608 | 0.009283473 |
| GO:0044421 | cellular_component | extracellular region part | 2 | 238 | 4.666666667 | 0.044499112 |
| GO:0008289 | molecular_function | lipid binding | 2 | 209 | 4.098039216 | 0.001496991 |
| GO:0006955 | biological_process | immune response | 2 | 183 | 3.588235294 | 4.77868E-09 |
| GO:0031982 | cellular_component | vesicle | 2 | 157 | 3.078431373 | 0.000291447 |
| GO:0009605 | biological_process | response to external stimulus | 2 | 154 | 3.019607843 | 0.022583766 |
| GO:0051674 | biological_process | localization of cell | 2 | 153 | 3 | 0.000112095 |
| GO:0009628 | biological_process | response to abiotic stimulus | 2 | 147 | 2.882352941 | 7.89268E-05 |
| GO:0009719 | biological_process | response to endogenous stimulus | 2 | 138 | 2.705882353 | 0.00121643 |
| GO:0016265 | biological_process | death | 2 | 131 | 2.568627451 | 0.015828871 |
| GO:0048037 | molecular_function | cofactor binding | 2 | 95 | 1.862745098 | 0.040617988 |
| GO:0030246 | molecular_function | carbohydrate binding | 2 | 83 | 1.62745098 | 0.042297629 |
| GO:0097367 | molecular_function | carbohydrate derivative binding | 2 | 64 | 1.254901961 | 0.000696878 |
| GO:0019882 | biological_process | antigen processing and presentation | 2 | 33 | 0.647058824 | 0.010538894 |
