# Supplementary material for: Analysis of Serum microRNA Expression Profiles and Comparison with Small Intestinal microRNA Expression Profiles in Weaned Piglets
Source: PLoS One. 2016 Sep 15;11(9):e0162776. doi: 10.1371/journal.pone.0162776 (PMC5025173; doi:10.1371/journal.pone.0162776)
Supplement: S6 Table — (DOCX) [file pone.0162776.s006.docx]

| KEGG Pathway | Count | % | P Value | FDR |
| --- | --- | --- | --- | --- |
| ssc05200:Pathways in cancer | 78 | 2.710215427 | 0.014805941 | 16.86806402 |
| ssc04062:Chemokine signaling pathway | 47 | 1.633078527 | 0.031262426 | 32.52166247 |
| ssc04514:Cell adhesion molecules (CAMs) | 38 | 1.320361362 | 0.029402019 | 30.89905881 |
| ssc04110:Cell cycle | 36 | 1.250868659 | 0.001779026 | 2.181131571 |
| ssc05215:Prostate cancer | 33 | 1.146629604 | 0.005789229 | 6.938294228 |
| ssc04640:Hematopoietic cell lineage | 31 | 1.077136901 | 0.025739492 | 27.59940384 |
| ssc03320:PPAR signaling pathway | 29 | 1.007644197 | 0.047763901 | 45.45507539 |
| ssc05220:Chronic myeloid leukemia | 27 | 0.938151494 | 0.00182881 | 2.24153364 |
| ssc04672:Intestinal immune network for IgA production | 25 | 0.868658791 | 0.004560914 | 5.50428575 |
| ssc05222:Small cell lung cancer | 25 | 0.868658791 | 0.020236133 | 22.36821116 |
| ssc05410:Hypertrophic cardiomyopathy (HCM) | 25 | 0.868658791 | 0.030415055 | 31.78699889 |
| ssc04662:B cell receptor signaling pathway | 24 | 0.833912439 | 0.060556375 | 53.86756099 |
| ssc05212:Pancreatic cancer | 24 | 0.833912439 | 0.060556375 | 53.86756099 |
| ssc05330:Allograft rejection | 22 | 0.764419736 | 0.039815446 | 39.54046851 |
| ssc04520:Adherens junction | 20 | 0.694927033 | 0.036132587 | 36.60489837 |
| ssc05221:Acute myeloid leukemia | 19 | 0.660180681 | 0.033982666 | 34.83121745 |
| ssc05214:Glioma | 18 | 0.625434329 | 0.074598937 | 61.71751877 |
| ssc00280:Valine, leucine and isoleucine degradation | 16 | 0.555941626 | 0.026113025 | 27.9424415 |
| ssc05219:Bladder cancer | 15 | 0.521195274 | 0.040558501 | 40.11738312 |
| ssc05310:Asthma | 14 | 0.486448923 | 0.096408107 | 71.50810643 |
| ssc05216:Thyroid cancer | 13 | 0.451702571 | 0.057133541 | 51.7421559 |
| ssc00071:Fatty acid metabolism | 13 | 0.451702571 | 0.092369557 | 69.89022452 |
| ssc00650:Butanoate metabolism | 11 | 0.382209868 | 0.045154416 | 43.57470521 |
| ssc03018:RNA degradation | 11 | 0.382209868 | 0.081227542 | 64.97855397 |
| ssc00410:beta-Alanine metabolism | 9 | 0.312717165 | 0.064082027 | 55.96655676 |

**S6 Table. Kyoto Encyclopedia of Genes and Genomes (KEGG) pathway annotations for potential miRNA targets.**
